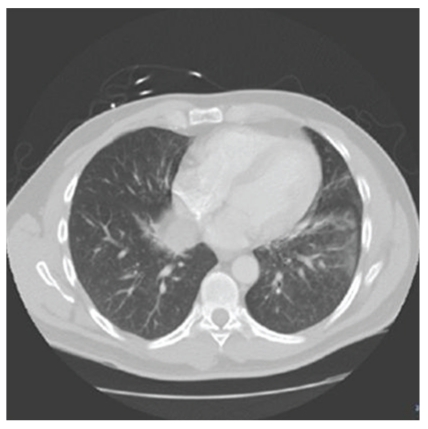# Airbag Pneumonitis

**DOI:** 10.1155/2010/498569

**Published:** 2010-10-17

**Authors:** Raghav Govindarajan, Gustavo Ferrer, Laurence A. Smolley, Eduardo Araujo Oliveira, Franck Rahaghi

**Affiliations:** Department of Pulmonary Medicine, Cleveland Clinic Florida, 2950 Cleveland Clinic Boulevard, Weston, FL 33331, USA

## Abstract

The widespread and mandatory use of airbags has resulted in various patterns of injuries and complications unique to their use. Airbags have been implicated in a spectrum of pulmonary conditions ranging from exacerbation of asthma, reactive airway diseases to new onset asthma. We report a case of inhalational chemical pneumonitis that developed after exposure to the airbag fumes.

## 1. Introduction

The 2001 United States national highway traffic safety administration report estimated that airbags reduce driver fatality by 12%–14% [1].The widespread and mandatory use of airbags has resulted in various patterns of injuries and complications unique to their use. Airbags have been implicated in a spectrum of pulmonary conditions ranging from exacerbation of asthma [2], reactive airway diseases [3] to new onset asthma [4]. Inhalational chemical pneumonitis is a rare complication associated with deployment of airbags [5]. 

## 2. Case Report

A 56-year-old Caucasian male with no significant past history was involved in a high-speed motor vehicle accident where by his car was hit at the rear end with rapid deceleration resulting in deployment of the airbag. The patient was exposed to airbag fumes within the closed confines of the car. The Emergency Medical Service reported a white powder in the patient mouth and nostrils. Subsequently he developed cough with scanty mucoid sputum. He had no shortness of breath, fever, chest pain, or palpitations. On physical examination, there were no obvious signs of injury, no tenderness of the ribs, or sternum. On auscultation, there were crackles in the left lower lobe. Patient was hemodynamically stable, afebrile, and saturating 96% in room air. There were no significant electrolyte abnormalities. Chest X-ray PA and lateral view revealed left lower lobe and retocardiac infiltrate. CT chest noncontrast revealed tree in bud opacities in the right middle lobe, left lingual. Ground glass opacity was also seen in the posterior basal segment of the left lower lobe (Figure 1). Sputum gram stain, sputum, and blood culture were noncontributory. Respiratory viral panel by PCR which included influenza A and B, respiratory synsytial virus, adenovirus, and parainfluenza 1, 2, and 3 were all negative. The followup noncontrast chest CT done in about 4 weeks revealed resolution of all the infiltrates. He has been following up with us for 6 months now and has not had any pulmonary issues.

## 3. Discussion

Sodium azide is a white colourless odourless crystalline powder [6]. It is a small hydrophilic compound and is the main propellant in the airbag system. Through a series of complex chemical reactions in the presence of high temperature, sodium azide is broken into nitrogen gas and sodium. It is the nitrogen gas which fills up the air bag and causes its insufflations [4]. The byproducts of this reaction also include particulate matter like salts of sodium. The particulates have been implicated in asthma and its exacerbation [2]. Inhalation of sodium azide in toxic levels has also been implicated in respiratory distress, pulmonary edema, and cardiopulmonary failure [6]. Our patient developed clinical signs, symptoms, and radiographic findings of pneumonitis following inhalational exposure to airbag propellants within the closed confines of his car. Differential considered were lung contusion, infective pneumonia. The fact that he developed symptoms immediately following inhalation; the CT findings were bilateral and involved the distal airways, and the workup for infective causes was negative favoured chemical pneumonitis. The main determinant in airway penetration and thus the site and type of lesion is particle size. Smaller particles can travel further down damaging the bronchiolar epithelium and alveoli. Sodium azide and some of the smaller sodium salts could be responsible for the chemical pneumonitis seen in our patient [5]. This pathological condition caused by sodium azide has not been described in vitro studies [6].

## 4. Conclusion

Although uncommon, the possibility of inhalational chemical pneumonitis should be considered in a patient with cough and pulmonary infiltrates involved in motor vehicle accident where the airbag gets deployed.

## Figures and Tables

**Figure 1:**